# Evolutionary history of plant hosts and fungal symbionts predicts the strength of mycorrhizal mutualism

**DOI:** 10.1038/s42003-018-0120-9

**Published:** 2018-08-16

**Authors:** Jason D. Hoeksema, James D. Bever, Sounak Chakraborty, V. Bala Chaudhary, Monique Gardes, Catherine A. Gehring, Miranda M. Hart, Elizabeth Ann Housworth, Wittaya Kaonongbua, John N. Klironomos, Marc J. Lajeunesse, James Meadow, Brook G. Milligan, Bridget J. Piculell, Anne Pringle, Megan A. Rúa, James Umbanhowar, Wolfgang Viechtbauer, Yen-Wen Wang, Gail W. T. Wilson, Peter C. Zee

**Affiliations:** 1 Department of Biology, University of Mississippi, University, MS 38677 USA; 2 Department of Ecology and Evolutionary Biology and Kansas Biological Survey, University of Kansas, Lawrence, KS 66045 USA; 3 Department of Statistics, University of Missouri, Columbia, MO 65201 USA; 4 Department of Environmental Science and Studies, DePaul University, Chicago, IL 60614 USA; 5 Laboratoire Évolution et Diversité Biologique, UMR5174 UPS – CNRS – IRD - ENSFEA, Université Toulouse III Paul Sabatier, Toulouse, France; 6 Department of Biological Sciences and Merriam-Powell Center for Environmental Research, Northern Arizona University, Flagstaff, AZ 86011 USA; 7 Department of Biology, University of British Columbia—Okanagan, Kelowna, BC V1V 1V7 Canada; 8 Departments of Biology and Mathematics, Indiana University, Bloomington, IN 47405 USA; 9 Department of Microbiology, Faculty of Science, King Mongkut’s University of Technology Thonburi, Bangkok, 10140 Thailand; 10 Department of Integrative Biology, University of South Florida, Tampa, FL 33620 USA; 11 Department of Land Resources and Environmental Sciences, Montana State University, 344 Leon Johnson Hall, Bozeman, MT 59717 USA; 12 Institute of Ecology and Evolution, University of Oregon, 335 Pacific Hall, Eugene, OR 97403 USA; 13 Department of Biology, New Mexico State University, Las Cruces, NM 88003 USA; 14 Department of Biology, College of Charleston, Charleston, SC 29424 USA; 15 Departments of Botany and Bacteriology, University of Wisconsin-Madison, Madison, WI 53706 USA; 16 Department of Biological Sciences, Wright State University, Dayton, OH 45435 USA; 17 Department of Biology, University of North Carolina, Chapel Hill, NC 27599 USA; 18 Department of Psychiatry and Neuropsychology, Maastricht University, 6200 MD Maastricht, Netherlands; 19 Natural Resource Ecology & Management, Oklahoma State University, Stillwater, OK 74078 USA

## Abstract

Most plants engage in symbioses with mycorrhizal fungi in soils and net consequences for plants vary widely from mutualism to parasitism. However, we lack a synthetic understanding of the evolutionary and ecological forces driving such variation for this or any other nutritional symbiosis. We used meta-analysis across 646 combinations of plants and fungi to show that evolutionary history explains substantially more variation in plant responses to mycorrhizal fungi than the ecological factors included in this study, such as nutrient fertilization and additional microbes. Evolutionary history also has a different influence on outcomes of ectomycorrhizal versus arbuscular mycorrhizal symbioses; the former are best explained by the multiple evolutionary origins of ectomycorrhizal lifestyle in plants, while the latter are best explained by recent diversification in plants; both are also explained by evolution of specificity between plants and fungi. These results provide the foundation for a synthetic framework to predict the outcomes of nutritional mutualisms.

## Abstract (web summary)

Jason Hoeksema et al. report a meta-analysis of the drivers of outcomes in mycorrhizal mutualisms across 646 plant–fungi combinations. They find that evolutionary history explains substantially more variation in the strength of mycorrhizal mutualisms than do ecological factors.

## Introduction

The last decade has seen the beginnings of a synthesis of community ecology and evolutionary biology^1^, as evolutionary history is increasingly used to explain ecological patterns and processes, such as community composition and assembly. However, new insights and greater predictive power may be achieved by quantifying the magnitudes and relative importance of evolutionary history versus contemporary ecological forces such as biotic and abiotic contextual factors^1^, not just for community assembly, but especially for ecologically relevant organismal traits, such as growth and population responses to species interactions^2^. A synthetic understanding of how evolutionary and ecological factors shape species traits and outcomes of foundational species interactions, such as nutritional symbioses, could allow modeling and prediction of the functional traits of communities that govern ecosystem processes, such as productivity and carbon storage^2^. For example, ecosystem-scale models of carbon and nitrogen cycling can now test the influence of traits of plant-microbial nutritional symbioses^3^, but synthetic data on these traits, and the factors driving their variability, are lacking. We sought to address this gap by asking how evolutionary and ecological factors shape plant growth responses to their ubiquitous nutritional symbioses with root-inhabiting mycorrhizal fungi.

Many plants and animals depend on symbioses for resource acquisition and defense. Among the most ancient and widespread of plant symbioses are the mycorrhizal associations of plant roots and fungi^4^. The majority of plant species, including most crops, associate with mycorrhizal fungi, and these symbioses influence terrestrial ecosystem responses to, and feedbacks with, changing environmental context^5,6^. Mycorrhizal symbioses can improve plant performance through enhanced soil nutrient uptake and other mechanisms, but net effects of fungal symbionts on host plants vary dramatically along a continuum from strong to weak mutualism, and even parasitism^7^. Despite the substantial consequences of these interactions for community function and ecosystem processes^5,8–10^, we lack a synthetic understanding of the evolutionary and ecological factors driving such variation for any nutritional symbiosis, including mycorrhiza, rhizobia, and corals^11^.

Ecological outcomes of plant-microbe symbioses have been intensively studied, but most research has focused on how contemporary ecological factors (biotic and abiotic contextual factors) drive plasticity within particular combinations of plants and microbes^11^. In many mycorrhizal symbioses, such context-dependency is important, particularly when increased availability to the plant of a limiting soil nutrient otherwise supplied by the fungus decreases plant benefits from the symbiosis^7,12^. Biotic context, including the presence of other microbes, can also drive contextual variation in plant responses to mycorrhizal fungi^13^. However, average plant response to mycorrhizal symbiosis apparently varies substantially among higher level taxa and clades, e.g., between warm-season C4 grasses and cool-season C3 grasses^14^, suggesting that evolutionary history may also exert an important influence on extant variation in the degree of mutualism.

At the coarsest level, mycorrhizal symbioses can be partitioned into several distinct association types, including arbuscular mycorrhizal and ectomycorrhizal, which differ in their evolutionary origins^4^. While there is a single origin of arbuscular mycorrhizal symbiosis in both plants and fungi, with subsequent losses and occasional reversions back to arbuscular mycorrhizal in the seed plants^4,15^, the ectomycorrhizal symbiosis stems from multiple, independent evolutionary origins in both plants and fungi^15–17^. We hypothesized that the differing genetic backgrounds and environmental contexts of the independent evolutionary origins of ectomycorrhizal symbiosis^4^ may have selected for different strengths of that mutualism.

While previous meta-analyses have explored the influences of particular ecological and evolutionary factors on focused sets of taxa^13,18–21^, we sought to quantify the joint influences of ecological contexts and evolutionary histories, including phylogenetic relationships of both hosts and symbionts. We did so by applying meta-analysis to a database (MycoDB) of plant responses to mycorrhizal fungi with unprecedented taxonomic breadth and sampling depth^22^. We tested the influence of early phylogenetic and recent diversification among plant species and fungal genera, non-independence of plant and fungal diversification (i.e., specificity of plant response to particular fungi due to non-independent evolution of plants and fungi); independent evolutionary origins of ectomycorrhizal symbiosis in plants and fungi; artificial selection through human domestication of plants; plant traits including functional groups and life history; and ecological factors, including nitrogen (N) and phosphorus (P) fertilization and the presence of additional non-mycorrhizal microbes.

We find that evolutionary history explains a substantial proportion of variation in plant responses to mycorrhizal fungi, and has different influences on outcomes of ectomycorrhizal versus arbuscular mycorrhizal symbioses. The former are best explained by the multiple evolutionary origins of ectomycorrhizal lifestyle in plants, while the latter are best explained by recent diversification in plants; both are also explained by evolution of specificity between plants and fungi. These results place evolutionary history alongside environmental context in development of a synthetic predictive framework for nutritional symbioses.

## Results

### Overall effect sizes and funnel plots

The overall weighted mean effect size, plant responsiveness to inoculation with mycorrhizal fungi (percent increase in plant growth due to mycorrhizal inoculation), was positive for both arbuscular mycorrhizal (AM-full: 65.7% ± 8.2 SE, AM-sub: 62.0% ± 5.9 SE) and ectomycorrhizal (80.3 ± 27.1 SE) symbiosis, indicating an average beneficial (~1.6–1.8-fold) effect of mycorrhizal inoculation on host plant biomass growth. None of the data sets had funnel plots with shapes indicating systematic publication bias^23,24^.

### Random effects of plants, fungi, and specificity

**Fig. 1:**
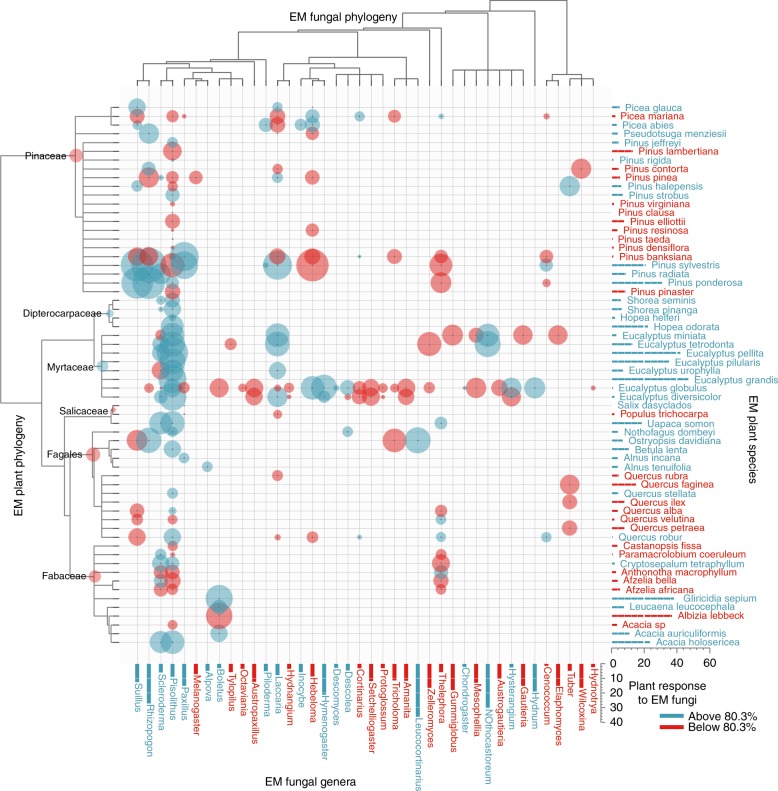
Heat map of plant response to ectomycorrhizal (EM) fungi across 190 combinations of EM plants and fungi (marked with bubbles). Bubble size indicates deviation of mean percent plant biomass response to EM fungi from the overall average of 80.3 (±27.1 SE) (blue above average, red below average), illustrating the effect of the plant phylogeny × fungal phylogeny interaction. Bars (plants right, fungi bottom) are marginal means across the bubble values. Node labels on the plant phylogeny indicate six independent evolutionary origins of EM symbiosis, with bubbles indicating magnitudes of lineage deviations from the overall mean (illustrating the effect of plant origin)

In ectomycorrhizal symbioses, the multiple, different evolutionary origins of ectomycorrhizal lifestyle in plants explained the most variation in plant response to ectomycorrhizal fungi (plant origin, partial *R*^2^ = 0.18; Table 1), resulting in substantial differences among plant clades in average responsiveness (Fig. 1). Plant response to ectomycorrhizal fungi was also partly explained by non-independent divergence across ectomycorrhizal plant and fungal phylogenies (plant phylogeny × fungal phylogeny interaction, partial *R*^2^ = 0.09), leading to specificity in plant lineage responses to ectomycorrhizal fungal lineages (Fig. 1, Table 1).

**Table 1:** Random-effect variance component estimates (and 95% CI^a^) from likelihood meta-analysis models in analyses of arbuscular mycorrhizal (AM) and ectomycorrhizal (EM) symbioses

Source	AM-sub data (*n* = 2398)	EM data (*n* = 1001)	Interpretation
Plant phylogeny	0.009 (0.0–0.15)	0.0 (0.0–0.07)	Phylogenetic heritability (“early” divergence) in plant hosts
Plant species	**0.15 (0.04–0.25),** ***R***^**2**^ = **0.24**^b^	0.0 (0.0–0.06)	Non-phylogenetic variation (“recent” divergence) among plant species or plasticity
Fungal phylogeny	0.0 (0.0–0.02)	0.0 (0.0–0.03)	Phylogenetic heritability (“early” divergence) in fungi
Fungal genus	0.0 (0.0–0.01)	0.0 (0.0–0.02)	Non-phylogenetic variation (“recent” divergence) among fungal genera or plasticity
Plant origin	N/A	**0.232 (0.01–1.5),** ***R***^**2**^ = **0.18**	Variation among seven EM host plant clades having independent evolutionary origins of EM lifestyle
Fungal origin	N/A	0.0 (0.0–0.03)	Variation among 24 EM fungal clades having independent evolutionary origins of EM lifestyle
Plant × fungal origin	N/A	0.01 (0.0–0.05)	Variation among 50 combinations of plant and fungal clades having independent evolutionary origins of EM lifestyle
Plant phylogeny × fungal phylogeny	0.0 (0.0–0.06)	**0.11 (0.01–0.16),** ***R***^**2**^ = **0.09**	Evolution of specificity between plant and fungal phylogenies
Plant phylogeny × fungal genus	**0.06 (0.0–0.09),** ***R***^**2**^ = **0.09**	0.0 (0.0–0.05)	Evolution of specificity between plant phylogeny and fungal genera
Plant species × fungal phylogeny	0.0 (0.0–0.05)	0.0 (0.0–0.09)	Evolution of specificity between plant species and fungal phylogeny
Plant species × fungal genus	0.0001 (0.0–0.06)	0.0 (0.0–0.03)	Recent divergence leading to specificity between plant species and fungal genera
Study ID	**0.10 (0.09–0.11),** ***R***^**2**^ = **0.15**	0.04 (0.03–0.05)	Residual between-studies variance
Control set	**0.16 (0.14–0.18),** ***R***^**2**^ = **0.24**	**0.15 (0.12–0.19),** ***R***^**2**^ = **0.12**	Non-independence among observations sharing a non-inoculated control
Paper	**0.15 (0.11–0.21),** ***R***^**2**^ = **0.24**	**0.65 (0.45–0.97),** ***R***^**2**^ = **0.51**	Non-independence among observations from the same primary paper

^a^95% CI is a profile likelihood confidence interval

^b^*R*^2^ is a partial conditional *R*^2^, which is the proportion of between-studies variance in effect size explained by a particular random effect. Bold print highlights likelihood variance components accounting for >5% of between-studies variance in likelihood analysis, for which *R*^2^ is shown

**Fig. 2:**
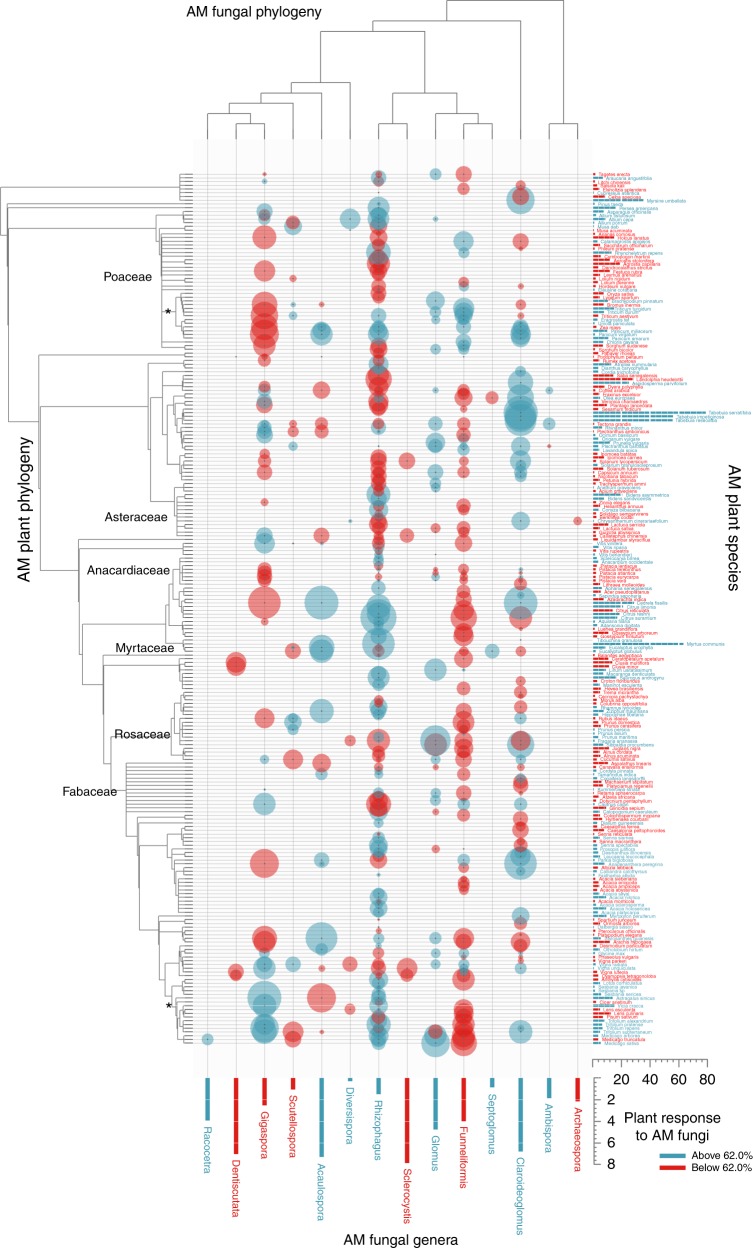
Heat map of plant response to arbuscular mycorrhizal (AM) fungi across 456 combinations of AM plants and fungi (marked with bubbles). Bubble size indicates deviation of mean percent plant biomass response to AM fungi from the overall average of 62.0 (±5.9 SE) (blue above average, red below average), illustrating the effect of the plant phylogeny × fungal genus interaction. Bars (plants right, fungi bottom) are marginal means across the bubble values. Plant families with five or more species in the data are labeled, and asterisks indicate the two plant clades (one each in Poaceae and Fabaceae) highlighted in the Discussion

By contrast, variation in plant response to arbuscular mycorrhizal fungi was largely explained by a combination of recent diversification among arbuscular mycorrhizal plant species (plant species, partial *R*^2^ = 0.24) and correlated evolution between early arbuscular mycorrhizal plant phylogenetic lineages and arbuscular mycorrhizal fungal genera (plant phylogeny × fungal genus interaction, partial *R*^2^ = 0.09), and not at all by early phylogenetic divergence in the arbuscular mycorrhizal fungi (Fig. 2, Table 1, AM-sub data).

Overall, likelihood and Bayesian estimates of random effect variance components were very similar. One difference was that for the four plant × fungus interaction effects, likelihood estimates tended to be consolidated in one larger value (plant phylogeny × fungal phylogeny for EM, plant phylogeny × fungal genus for AM-sub) with the other three estimated near zero, whereas Bayesian estimates were distributed among three (EM) or four (AM-sub) of the four interactions (Supplementary Table 1). In both AM-sub and EM analyses, however, the sum totals of variance components for the four plant × fungus interaction effects were very similar between likelihood and Bayesian estimates. Results were qualitatively insensitive to which method was used to impute missing values of effect size variance, although estimated magnitudes of random-effect variance components were sometimes smaller and had greater uncertainty when multiple imputation (HotDeck_NN) was used (Supplementary Table 2).

### Fixed effects of ecological and experimental context

**Fig. 3:**
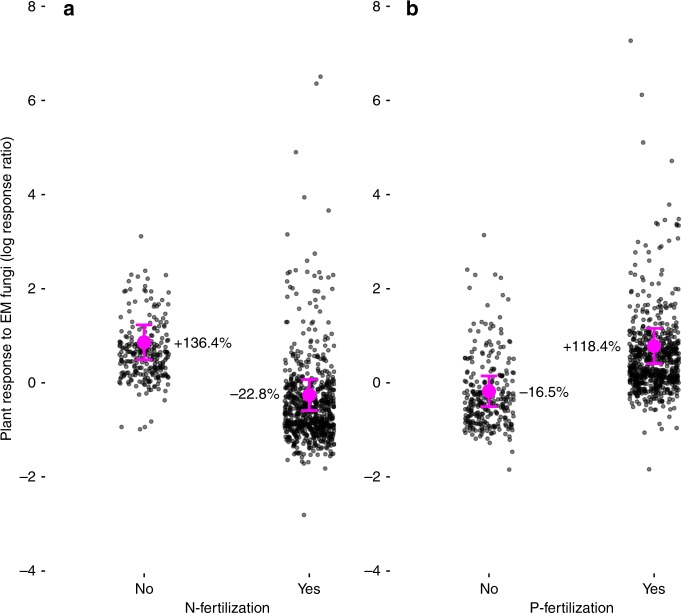
Influence of nitrogen (N, **a**) and phosphorus (P, **b**) fertilization on plant biomass response to inoculation with ectomycorrhizal (EM) fungi. Vertical axis is log response ratio (LRR) of mean inoculated plant biomass to mean non-inoculated plant biomass. Marginal means and SE are in magenta, and raw data are adjusted for effects of the three other fixed effects in the model and jittered for display to reduce overplotting. Both marginal means and adjusted data were derived from the best mixed model for EM symbiosis according to likelihood model selection, fit with restricted maximum likelihood (REML). Labels of marginal means are percent increase or decrease of plant growth due to mycorrhizal inoculation, transformed from LRR as 100 × (e^LRR^-1)

For ectomycorrhizal symbiosis, the best model from both ML and REML model selection included the fixed effects of N-fertilization, P-fertilization, Sterilization, and Microbial Control, and had a marginal *R*^2^ of 0.055, indicating that fixed effects explained about 5% of variation in plant response to ectomycorrhizal fungal inoculation. REML model selection analyses determined that two of the fixed-effect predictors had relative variable importance (RVI) well above 0.5: N-fertilization (0.78) and P-fertilization (0.73). Adding N-fertilizer was associated with a decreased plant response, while adding P-fertilizer was associated with increased plant response (Fig. 3). The other four fixed effects had RVI values near or <0.5 (sterilization: 0.52, microbial control: 0.51, plant functional group: 0.22, location: 0.17). ML model selection results were qualitatively similar to those of REML model selection (Supplementary Fig. 1), and Bayesian *P*-values indicated significance only for N-fertilization, P-fertilization, and sterilization.

In the best models of arbuscular mycorrhizal symbiosis from REML model selection, no fixed effects were included, and no fixed factors were significant according to Bayesian *P*-values, suggesting that fixed effects of context and plant traits explained none of the between-studies variance in plant response to arbuscular mycorrhizal inoculation. Under REML model selection on the AM-full data, all 13 fixed effects had RVI <0.4. The best model from ML model selection for arbuscular mycorrhizal symbiosis contained only the fixed effects of sterilization, P-fertilization, and inoculum complexity (see also Supplementary Fig. 2), although there were 17 other models within 2 AICc units of the best model and the marginal *R*^2^ was 0.012, indicating minimal explanatory value of fixed effects.

## Discussion

Among ectomycorrhizal symbioses, plant response to ectomycorrhizal fungi was most strongly explained by the multiple, different evolutionary origins of ectomycorrhizal symbiosis in plants. These origins have left a legacy of divergence in average plant responses to ectomycorrhizal fungi, from >50% below the average response in some clades (e.g., Fabaceae), to well above the average response in other clades (e.g., Myrtaceae) (Fig. 1). This result suggests that evolutionary convergence of interaction phenotypes (e.g., the general morphology of ectomycorrhizal symbiosis) does not always lead to uniformity in ecological function^25^. In this case, it supports the hypothesis that differing genetic backgrounds and/or environmental contexts during the independent evolutionary origins of ectomycorrhizal symbiosis^4^ may have selected for different strengths of that mutualism. For example, selection may have favored reduced responsiveness to ectomycorrhizal symbiosis in plant lineages such as Fabaceae that were already engaged in nitrogen-fixing rhizobial symbiosis with bacteria when the ectomycorrhizal fungal symbiosis arose in Fabaceae^15,26^. Engagement in this N-fixing symbiosis with bacteria may have reduced both demand for soil-derived nitrogen and benefits from ectomycorrhizal symbiosis, compared to plant clades that were not engaged in rhizobial symbiosis when ectomycorrhizal symbiosis arose.

Plant response to ectomycorrhizal fungi was also explained by non-independent evolution across ectomycorrhizal plant and ectomycorrhizal fungal phylogenies, leading to specificity of plant lineage responses to ectomycorrhizal fungal lineages (Fig. 1). For example, responsiveness of plants in the family Myrtaceae to the fungal lineage including *Pisolithus* and *Scleroderma* is more positive than their response to the fungal lineage including *Suillus* and *Rhizopogon*, but the opposite is true for plants in the family Pinaceae (Fig. 1). Such specificity may impact coexistence among species in both plant and fungal communities through feedback dynamics^27^, and provides guidance for selecting appropriate mycorrhizal fungi for use in forestry, horticulture, and restoration applications. It is important to note that a history of reciprocal coevolutionary selection cannot necessarily be inferred from non-independent evolution of a trait on host and symbiont phylogenies, as reciprocal selection is not required to generate such patterns^28^, and our phylogenies actually modeled drift evolution. Extending phylogenetic mixed meta-analysis models to explore scenarios of selection would be a desirable future direction.

In contrast to ectomycorrhizal symbiosis, variation in plant response to arbuscular mycorrhizal fungi was primarily explained by recent diversification among plants (Table 1). Previous studies found evidence for plant phylogenetic history driving plant response to arbuscular mycorrhizal symbiosis^19,29^, but were focused on more limited sets of taxa and, in particular, did not test the influence of fungal phylogeny or interactions between plant and fungal phylogeny; the latter absorbed plant phylogenetic effects in our models (Supplementary Table 1, compare AM-full to AM-sub). This result highlights the importance of considering evolutionary history on both sides of species interactions when seeking to explain variability in species interactions or their underlying traits.

In this case, non-independent evolution between arbuscular mycorrhizal plant phylogenetic lineages and arbuscular mycorrhizal fungal genera (Fig. 2, Table 1) explained variation in plant response to arbuscular mycorrhizal fungi. This result implies that extant specificity in how plants respond to arbuscular mycorrhizal fungi has resulted from recently evolved differences among arbuscular mycorrhizal fungal genera in how they affect growth responses of plants in particular phylogenetic lineages (Fig. 2). For example, responsiveness of plants in the Fabaceae clade containing *Trifolium* and *Medicago* to the fungal genus *Gigaspora* is more positive than their response to *Funneliformis*, but the opposite is true for plants in the Poaceae clade containing *Triticum*, *Sorghum*, and *Panicum* (Fig. 2).

We found no evidence that phylogenetic diversification in arbuscular mycorrhizal fungi accounts for contemporary patterns of plant responsiveness to those fungi. Previous studies have found such evidence^30^, but were based on more limited taxon sampling, and did not simultaneously explore the influence of plant diversification. It is likely that divergence among fungal species within genera, and/or among fungal populations or clones within species, is ongoing; if so, this would further support our conclusion of recent evolutionary divergence as a driver of plant growth responses to arbuscular mycorrhizal fungi. Indeed, a recent field experiment with 56 arbuscular mycorrhizal fungal isolates from 17 genera found that a large proportion of variation in promotion of host plant growth was among different isolates of the same arbuscular mycorrhizal fungal species^31^.

Ecological contextual factors included in our models did not explain variation in plant responses to arbuscular mycorrhizal fungi and had limited explanatory power for ectomycorrhizal symbiosis. In particular, plant response to ectomycorrhizal fungi was negatively affected by N-fertilization (Fig. 3), which is consistent with findings of a previous meta-analysis^13^ and with observations that ectomycorrhizal colonization and diversity are negatively affected by atmospheric N deposition^32^. By contrast, plant responses to ectomycorrhizal fungi were positively associated with P-fertilization (Fig. 3), which could be linked to increased efficiency of P transfer to host plants by ectomycorrhizal fungi with increased P availability in soils^33^, and/or increased value of ectomycorrhizal fungal provisioning of N to plants when P becomes less limiting. Although context-dependency can substantially shape the responses of particular plant species to mycorrhizal fungi^7,12^, our analysis suggests that at a broad comparative scale its influence may be small, relative to evolutionary history. This observation supports the general hypothesis of increasing phylogenetic conservatism of traits with increasing phylogenetic scale^2^.

An important caveat to our conclusions regarding ecological context, however, is that we were only able to explore a limited set of such factors here. For example, ambient light availability was not included, but may limit plant benefits to arbuscular mycorrhizal and ectomycorrhizal fungi, with plant growth responses to fungi depressed under low ambient light^7,12^. In addition, we captured some potential influence of soil nutrients by testing the influence of N- and P-fertilization; however, actual nutrient concentrations in background soil are likely also important. Unfortunately, background soil nutrient concentrations and ambient light availability are reported so inconsistently in the primary experimental literature that they cannot be included in a large-scale analysis with factors that are reported much more frequently. Additionally, most experiments analyzed here took place in the absence of biotic interactions, such as herbivory and disease, that can influence benefits of mycorrhizal symbioses in natural systems. Finally, our analyses were applied to only a subset of all the plant-fungal combinations occurring nature. A more complete picture of the relative importance of ecological context versus evolutionary history awaits future analyses of expanded data sets that are enriched for contextual factors not tested here.

Our results shed new light on variation in ecological outcomes of mycorrhizal symbioses, highlighting the importance of evolutionary history. We suggest that these results are relevant to other types of nutritional symbioses such as rhizobia and corals, which involve trade of resources including photosynthates and have also been more thoroughly explored for their context-dependency than for the influence of evolutionary history^11^. Although previous discussions of variability in mycorrhizal symbioses have focused on the importance of environmental contextual factors such as nutrient availability, we have shown here that evolutionary history plays a large role in driving variability in contemporary outcomes of these interactions. Plant growth responses to both arbuscular mycorrhizal and ectomycorrhizal symbioses are shaped by evolved specificity, and plant responses to ectomycorrhizal symbiosis remain a legacy of the original independent evolutionary origins of ectomycorrhizal symbiosis in plants. Mycorrhizal symbioses may be the most intensively studied nutritional symbiosis on earth, but the wide array of interaction outcomes, which range from mutualism to parasitism, have largely defied synthetic explanation to date. Our results provide building blocks for a synthetic eco-evolutionary framework predicting outcomes of nutritional symbioses, and suggest that evolutionary history must be considered alongside ecological factors.

## Methods

### Overview and data

We conducted separate phylogenetic mixed-model meta-analyses for arbuscular mycorrhizal and ectomycorrhizal symbioses using the most recent version of the MycoDB database and associated fungal and plant phylogenetic trees (MycoDB_version4, FungalTree_version2, and PlantTree_version2), which contain data on plant biomass responses to inoculation with arbuscular mycorrhizal and ectomycorrhizal fungi, biotic and abiotic contextual factors varying among trials, species traits, and evolutionary origins and phylogenetic relationships of plant host species and fungal symbiont genera^22^. Fungal identities in MycoDB are coded to genus and not species because in many cases, recent revisions in fungal systematics make assigning taxa in older papers to new groups problematic, and because fungal species names are inconsistent among publications and thus difficult to definitively link to particular taxa. Compared to previous versions of the database, MycoDB_version4 and the associated phylogenetic trees (FungalTree_version2 and PlantTree_version2) exclude observations on a small number of possibly non-mycorrhizal or misidentified fungal taxa, update or correct nomenclature and/or phylogenetic placement of some plant and fungal taxa, and add new variables on independent evolutionary origins of mycorrhizal lifestyle in ectomycorrhizal plants and fungi. Our analyses were conducted on three subsets of MycoDB_version4: one (AM-full) in which plants were inoculated with arbuscular mycorrhizal fungi belonging to one or more fungal genera (2984 studies across 293 plant species and 14 fungal genera from 359 publications), a second (AM-sub) that was a subset of AM-full in which plants were only inoculated with a single arbuscular mycorrhizal fungal genus (2398 studies across 234 plant species, 14 fungal genera, and 456 unique plant-fungus combinations, from 297 publications), and a third (EM-sub, hereafter EM) in which plants were inoculated with ectomycorrhizal fungi belonging to only a single fungal genus (1001 studies across 62 plant species, 40 fungal genera, and 190 unique plant-fungus combinations, from 83 publications). Very few studies were on plants inoculated with species from more than one genus of ectomycorrhizal fungi, or with both arbuscular mycorrhizal and ectomycorrhizal fungi, so those studies were not included in our analyses. Because the studies in the AM-sub and EM data sets used inoculation with a single fungal genus, analyses of those data could include fungal genus, fungal phylogeny, and plant × fungal interactions in meta-analytic models. For a brief discussion of how the scope of inference from meta-analysis of MycoDB may be affected by the nature of the studies included, see Supplementary Methods.

### Calculation of effect size and estimated sampling variance

For all analyses, the response variable was the effect size of plant biomass response to mycorrhizal inoculation, expressed as a log response ratio^34^:

$${\mathrm LRR} = {\mathrm{ln}}\left[ {\frac{{\bar x_{{\mathrm{inoc}}}}}{{\bar x_{{\mathrm{ctrl}}}}}} \right],$$

where $\bar x_{{\mathrm{inoc}}}$ and $\bar x_{{\mathrm{ctrl}}}$ are mean plant biomass (total biomass if available, otherwise shoot biomass) in an inoculated treatment and a non-inoculated control, respectively. Positive values of this metric indicate beneficial effects of mycorrhizal inoculation and negative values indicate detrimental effects of mycorrhizal inoculation. When individual studies reported measures of dispersion in addition to sample sizes and means for inoculated and control groups, the sampling variance of LRR was estimated with:

$$\hat \sigma ^2 = \frac{{{\rm SD}_{{\mathrm{inoc}}}^2}}{{n_{{\mathrm{inoc}}} \times \bar x^2_{{\mathrm{inoc}}}}} + \frac{{{\rm SD}_{{\mathrm{ctrl}}}^2}}{{n_{{\mathrm{ctrl}}} \times \bar x^2_{{\mathrm{ctrl}}}}},$$

where ${\rm SD}_{{\mathrm{inoc}}}$ and ${\rm SD}_{{\mathrm{ctrl}}}$ are the standard deviation, and $n_{{\mathrm{inoc}}}$ and $n_{{\mathrm{ctrl}}}$ the number of replicates in the inoculated treatment and non-inoculated control groups, respectively^34^. However, when studies failed to report standard deviations or other metrics that could be used to compute it, we used the same equation for the variance, but with the coefficient of variation (the ratio ${\rm SD}/\bar x$) replaced by its median value from those studies that did report SDs (4.6% of studies in AM-full, 3.5% of studies in AM-sub, and 21.2% of studies in EM). This imputation was performed separately for each data set (AM-full, AM-sub, and EM). We also explored the robustness of results to alternative imputation methods (see Supplementary Methods).

### Random factors included in meta-analysis models

All models of both arbuscular mycorrhizal and ectomycorrhizal symbiosis potentially included these 11 random effects: plant phylogeny, plant species, fungal phylogeny, fungal genus, plant phylogeny × fungal phylogeny interaction, plant phylogeny × fungal genus interaction, plant species × fungal phylogeny interaction, plant species × fungal genus interaction, study ID, control set, and paper. The first four of those random effects correspond to the phylogenetically heritable and non-heritable variance components (for plants and fungi, respectively) of the phylogenetic mixed model described by Housworth et al.^35^. For example, plant phylogeny represents phylogenetically heritable variation, i.e., early evolutionary divergence in the trait, and plant species represents non-heritable variation, including rapid recent evolution in response to the environment as well as plasticity. The first eight random effects correspond to the eight components contributing to host-symbiont covariance in the two-phylogeny comparative trait evolution model of Hadfield et al.^36^. The six random effects involving fungi were not included in analyses of the AM-full data, since that data subset contained observations in which plants were inoculated with more than one fungal genus. Study ID was a unique identifier for each observation (i.e., effect size); its inclusion specifies the conventional mixed-effect meta-analytic model with random intercepts at the observation level, and its variance component corresponds to the residual between-studies variance (as modeled in more conventional random-effects meta-analyses and typically referred to as the between-studies variance). Control set and paper were included to account for potential non-independence among multiple effect sizes that were calculated using the same control group (i.e., non-inoculated mean plant biomass) or came from the same original scientific paper, respectively. Random effects for plant phylogeny and fungal phylogeny were associated with phylogenetic correlation matrices corresponding to the plant and fungal phylogenies. These phylogenetic correlation matrices assumed full Brownian motion evolution (lambda-fitted with *λ* = 1.0) since they contain the relative pairwise phylogenetic branch-length distance between species (for plants) or genera (for fungi)^37^. For interactions involving at least one phylogenetic random effect (plant phylogeny × fungal phylogeny, plant species × fungal phylogeny, and plant phylogeny × fungal genus), associated phylogenetic correlation interaction matrices were created by calculating the tensor products of the two corresponding correlation matrices^38^. Models of ectomycorrhizal symbiosis additionally included three random effects—plant origin, fungal origin, and plant × fungal origin—that coded for unique evolutionary origins of an ectomycorrhizal lifestyle among ectomycorrhizal plant lineages, ectomycorrhizal fungal lineages, and combinations of ectomycorrhizal plant and ectomycorrhizal fungal lineages, respectively. For details of how these evolutionary origins were determined, see Supplementary Methods.

### Fixed factors included in meta-analysis models

Saturated mixed models, i.e., models containing all possible factors, for analyses of both arbuscular mycorrhizal and ectomycorrhizal symbioses all contained the main effects of the following six fixed-effect predictors: N-fertilization (whether or not nitrogen fertilizer was added to background soil), P-fertilization (whether or not phosphorus fertilizer was added to background soil), sterilization (whether or not background soil was sterilized), microbial control (whether or not a filtrate of non-mycorrhizal microbes was added to all the background soil or a filtrate from the inoculum was added to non-inoculated soil), Location (whether the experiment was performed in the lab, i.e., greenhouse or growth chamber, or in the field), and Plant Functional Group (AM: C4 grass, C3 grass, nitrogen-fixing forb, non-nitrogen-fixing forb, nitrogen-fixing woody, or non-nitrogen-fixing woody; EM: nitrogen-fixing woody or non-nitrogen-fixing woody). The data for arbuscular mycorrhizal symbiosis allowed us to test two additional fixed-effect predictors on arbuscular mycorrhizal plant traits: plant life history (annual/biennial or perennial) and domestication (whether the host plant was a wild variety, a forage crop, or a domesticated variety). Finally, the AM-full data subset allowed us to test an additional fixed-effect predictor, Inoculum Complexity (single fungal species, multiple fungal species, or whole soil inoculum). Additional details on construction of these nine fixed-effect predictors can be found in Chaudhary et al.^22^.

Replication in our updated version of MycoDB was sufficient to test some two-way interactions between fixed-effect predictors, unlike a previous analysis of an earlier version of MycoDB^13^. To simplify the candidate set of models, two-way interactions between pairs of fixed-effect predictors were selected for analysis only when we could conceive hypotheses on how they would influence the response variable^39^, and when the structure of the data allowed testing of those interactions. The latter criterion was never satisfied for two-way interactions of interest for the ectomycorrhizal symbiosis. In the saturated model for arbuscular mycorrhizal symbiosis, the following two-way interactions were included: N-fertilization × P-fertilization, N-fertilization × plant functional group, P-fertilization × plant functional group, and sterilization × microbial control.

### Estimating the importance of fixed-effect predictors

Because meta-analysis data sets are observational with respect to differences in study-level fixed-effect predictors, null hypothesis tests of particular fixed-effect predictors can be influenced by correlations among predictors and can vary among models containing different combinations of predictors^39,40^. Indeed, in preliminary analyses we found *P*-values for particular fixed-effect predictors to vary substantially among models containing different sets of fixed effects. Thus, rather than rely on null hypothesis testing for stepwise determination of a single reduced model of fixed effects, we used likelihood model fitting and conducted model selection guided by information criteria (specifically, Akaike’s Information Criterion corrected for small sample sizes, or AICc^41^) to explore the relative importance of fixed-effect predictors among subsets of models, all of which contained all of the random effects estimated as non-zero in preliminary fitting of saturated mixed or pure random-effect models. In addition, we checked the sensitivity of these results to the model fitting approach by using Bayesian model fitting with saturated models containing all random and fixed effects, and examining the 95% credible interval and Bayesian *P*-values for fixed effects to determine their significance relative to an alpha of 0.05. For further details of how likelihood and Bayesian methods were used to determine the importance of fixed-effect predictors, see Supplementary Methods.

### Estimating magnitudes of random effect variance components

To characterize random effects, we used restricted maximum-likelihood estimation (REML) to fit models that were determined to be the best (with respect to which fixed effects were included) according to AICc-based model selection, as described above. From these models, the influences of random effects were ascertained by examining estimated magnitudes of associated variance components, along with their associated profile likelihood confidence intervals, which were estimated using the *confint()* function of the R package *metafor*. Likelihood profiles were obtained for all variance components to confirm their identifiability and convergence to the global optimum in each dimension^42^. To estimate the variance explained by particular random effects, we calculated a partial conditional *R*^2^ for each of those random effects in the AICc-best likelihood model, using the same equation as that for Nakagawa and Schielzeth’s^43^ conditional *R*^2^ for all random and fixed effects combined, but modified to include only the variance component for a particular random effect in the numerator, rather than variance components for all random and fixed effects. For arbuscular mycorrhizal symbiosis, because the AM-sub data allowed inclusion of variance components for fungi and for plant × fungus interactions, we focus on random effects estimated from the AM-sub data, although results from the AM-full data are presented for comparison (Supplementary Table 1). As a check on the sensitivity of results to the model fitting approach, we also estimated random effects using a Bayesian approach (see Supplementary Methods for details) for comparison with the results from likelihood estimation.

To obtain an overall estimate of the weighted mean effect size (LRR), we fit a pure random-effects model with all random effects for each data set separately, using REML estimation. Best linear unbiased predictors (BLUPs) for random effects involving plant species, fungal genera, and evolutionary origins were estimated from the AICc-best likelihood models using the *ranef*() function in *metafor*. These BLUPs, representing deviations from the overall weighted mean effect size of plant response to mycorrhizal fungi, were used to quantify and visualize random effects. For example, to quantify variation in the LRR among independent evolutionary origins of ectomycorrhizal host plants, we used the Plant Origin BLUPs (see node labels and node bubbles in Fig. 1). One of the plant evolutionary origins, Phyllanthaceae, was represented in the data by only a single plant species (*Uapaca somon*), so it was not labeled on Fig. 1. To visualize the random effects of ectomycorrhizal plant phylogeny × fungal phylogeny (plant/fungus bubbles in Fig. 1) and arbuscular mycorrhizal plant phylogeny × fungal genus (plant/fungus bubbles in Fig. 2), we generated 2-phylogeny heat maps of the corresponding BLUPs using the *input_trees*() and *plot_trees*() functions in the *dualingTrees* package of R (available at github.com/jfmeadow/dualingTrees-pkg). Fungal genus and plant species means, calculated as marginal means across plant-fungus combinations, are shown at the bottom and right sides of the heat maps, respectively. For arbuscular mycorrhizal and ectomycorrhizal symbiosis, these BLUPs were taken from analysis of the best models of the AM-sub and EM data, respectively. For ease of interpretation, all means and BLUPs of effect size (log response ratio, or LRR) were transformed (100 × (e^LRR^-1)) to represent percent change in plant biomass growth in response to mycorrhizal inoculation.

### Data availability

The data sets generated and analyzed during the current study (MycoDB_version4, FungalTree_version2, and PlantTree_version2) are available in the Dryad Digital Repository (https://datadryad.org//resource/doi:10.5061/dryad.723m1.4)^44^. The data repository in Dryad also includes data from the published data descriptor for MycoDB^45^.

### Code availability

The original R code written for the analyses presented here is available in the Dryad Digital Repository (https://datadryad.org//resource/doi:10.5061/dryad.723m1.4)^44^.

## Electronic supplementary material

Supplemental Information
