# Author Correction: Evolutionary history of plant hosts and fungal symbionts predicts the strength of mycorrhizal mutualism

**DOI:** 10.1038/s42003-018-0143-2

**Published:** 2018-09-06

**Authors:** Jason D. Hoeksema, James D. Bever, Sounak Chakraborty, V. Bala Chaudhary, Monique Gardes, Catherine A. Gehring, Miranda M. Hart, Elizabeth Ann Housworth, Wittaya Kaonongbua, John N. Klironomos, Marc J. Lajeunesse, James Meadow, Brook G. Milligan, Bridget J. Piculell, Anne Pringle, Megan A. Rúa, James Umbanhowar, Wolfgang Viechtbauer, Yen-Wen Wang, Gail W. T. Wilson, Peter C. Zee

**Affiliations:** 1 Department of Biology, University of Mississippi, University, MS 38677 USA; 2 Department of Ecology and Evolutionary Biology and Kansas Biological Survey, University of Kansas, Lawrence, KS 66045 USA; 3 Department of Statistics, University of Missouri, Columbia, MO 65201 USA; 4 Department of Environmental Science and Studies, DePaul University, Chicago, IL 60614 USA; 5 Laboratoire Évolution et Diversité Biologique, UMR5174 UPS - CNRS - IRD - ENSFEA, Université Toulouse III Paul Sabatier, Toulouse, France; 6 Department of Biological Sciences and Merriam-Powell Center for Environmental Research, Northern Arizona University, Flagstaff, AZ 86011 USA; 7 Department of Biology, University of British Columbia—Okanagan, Kelowna, BC V1V 1V7 Canada; 8 Departments of Biology and Mathematics, Indiana University, Bloomington, IN 47405 USA; 9 Department of Microbiology, Faculty of Science, King Mongkut’s University of Technology Thonburi, Bangkok, 10140 Thailand; 10 Department of Integrative Biology, University of South Florida, Tampa, FL 33620 USA; 11 Department of Land Resources and Environmental Sciences, Montana State University, 344 Leon Johnson Hall, Bozeman, MT 59717 USA; 12 Institute of Ecology and Evolution, University of Oregon, 335 Pacific Hall, Eugene, OR 97403 USA; 13 Department of Biology, New Mexico State University, Las Cruces, NM 88003 USA; 14 Department of Biology, College of Charleston, Charleston, SC 29424 USA; 15 Departments of Botany and Bacteriology, University of Wisconsin-Madison, Madison, WI 53706 USA; 16 Department of Biological Sciences, Wright State University, Dayton, OH 45435 USA; 17 Department of Biology, University of North Carolina, Chapel Hill, NC 27599 USA; 18 Department of Psychiatry and Neuropsychology, Maastricht University, 6200 Maastricht, Netherlands; 19 Natural Resource Ecology & Management, Oklahoma State University, Stillwater, OK 74078 USA

Correction to: *Communications Biology* 10.1038/s42003-018-0120-9, published online 16 August 2018

In the original published version of the article, the description of the fixed-effect predictor Inoculum Complexity presented in the Methods was incorrect. The incorrect description given was: “single fungal genus, multiple fungal genera, or whole soil inoculum”. The correct description is: “single fungal species, multiple fungal species, or whole soil inoculum”. The error does not affect any of the results presented in the paper. The correction has been made to the HTML and PDF versions of the paper.

