# Supplementary material for: Evolutionary history of plant hosts and fungal symbionts predicts the strength of mycorrhizal mutualism
Source: Commun Biol. 2018 Aug 16;1:116. doi: 10.1038/s42003-018-0120-9 (PMC6123707; doi:10.1038/s42003-018-0120-9)
Supplement: Supplementary file 1 — Supplemental Information [file 42003_2018_120_MOESM1_ESM.pdf]

Supplementary Methods

Inferences from meta-analysis

Conclusions from meta-analyses on diverse species interactions, such as those presented here, are only as general as the sample of taxa present in the data. The most abundant ectomycorrhizal fungi in MycoDB belong to genera that are relatively easily cultured (e.g., *Suillus* & *Laccaria*) and/or easily inoculated onto plant roots using spores (e.g., *Rhizopogon* and *Pisolithus*), and many combinations of plants and fungi found engaging in symbiosis in nature are missing altogether from the database. The more complete is this matrix of plant-fungus combinations in the data, the more representative of nature the results of such meta-analyses will be. In addition, we have only analyzed plant biomass growth responses, and different insights might be drawn from analysis of plant performance parameters such as survival or seed production. Moreover, most ectomycorrhizal inoculation experiments are necessarily performed on woody plant seedlings. Despite the importance of seedling stages as a crucial bottleneck in the demography of many tree populations, it is possible that adult tree responses to ectomycorrhizal fungi are affected differently than seedlings by the evolutionary and contextual factors analyzed here. Ectomycorrhizal plants may also respond differently to diverse suites of ectomycorrhizal fungi, which they typically encounter in the field, compared to the small number of fungal taxa used in most studies in our ectomycorrhizal data. Contextual factors varying among published papers and not yet included in a database due to limited reporting, or data on life history traits such as successional status, could potentially explain additional variation in plant responses to mycorrhizal fungi[1].

Coding of evolutionary origins of ectomycorrhizal (EM) lifestyle

Independent evolutionary origins of the ectomycorrhizal lifestyle among host plants were assigned to the Plant Origin variable based on phylogenetic analyses of mycorrhizal status across the plant kingdom[2,3]. Our ectomycorrhizal data contained observations on nine plant families, all of which besides Pinaceae belong to the Rosid clade of Angiosperms. It is clear that Pinaceae has an evolutionary origin of an ectomycorrhizal lifestyle independent of the Rosids, although there is uncertainty about whether the ancestral state among Rosids was ectomycorrhizal or arbuscular mycorrhizal[2]. Thus, we tested two alternative Plant Origin variables, one of which assumed a single origin of ectomycorrhizal lifestyle within the Rosids, and thus two overall in our data (Pinaceae and Rosids), and another of which assumed multiple origins of ectomycorrhizal lifestyle within the Rosids, and thus seven in our data (Pinaceae, Dipterocarpaceae, Fabaceae, Fagales (including Betulaceae, Fagaceae, and Nothofagaceae), Myrtaceae, Phyllanthaceae, and Salicaceae). In preliminary analyses, we found that only the latter version of Plant Origin had explanatory value, so we only present results using the latter.

Independent evolutionary origins of an ectomycorrhizal lifestyle among the fungi were also estimated using published phylogenetic studies. We used the independent evolutionary origins hypothesized by Tedersoo et al.[4] as a starting point. Fungal genera not included in the analysis of Tedersoo et al.[4] were assigned shared or independent origins according to multiple phylogenic studies[5-7]. If a fungal genus belonged to a monophyletic ectomycorrhizal clade or had an immediate ectomycorrhizal sister clade, the genus was assigned an origin shared with the ectomycorrhizal group. If no closely related ectomycorrhizal lineage was reported, the genus was assigned an independent origin. We then tested the independent origins hypothesized by Tedersoo et al.[4] using ancestral state analysis with a gain:loss model that assumed an equal likelihood of gain and loss of the ectomycorrhizal lifestyle, which is a conservative assumption

with respect to independent origins of ectomycorrhizal lifestyle, contrasting with the approach used by Tedersoo et al.[4], which assumed no reversals from ectomycorrhizal to other lifestyles. Three other studies[2,8,9] were also consulted to detect inconsistencies in hypothesized ectomycorrhizal origins among phylogenetic analyses. Such inconsistencies were analyzed individually using published phylogenetic studies of those particular lineages[9-17] to reconstruct the ancestral mycorrhizal status (EM or not) and determine whether the genera shared the same ectomycorrhizal origin, using a maximum parsimony criterion. Tedersoo et al.'s[4] hypotheses for independent ectomycorrhizal origins were rejected if an alternative model with fewer niche switching steps found greater support. Despite our conservative approach, we found only one clade in which a hypothesis of fewer ectomycorrhizal origins was supported in comparison to the hypotheses of Tedersoo et al.[4]; our analysis favored only one ectomycorrhizal origin for *Hydnotrya* and *Tuber*. However, the explanatory value of the Fungal Origin variable was identical regardless of how this clade was treated, so we used a Fungal Origin variable with two origins in the *Hydnotrya*/*Tuber* clade and a total of 24 ectomycorrhizal origins. We also created a Plant × Fungus Origin variable, the levels of which were the 50 unique combinations of ectomycorrhizal plant and ectomycorrhizal fungal origin present in the ectomycorrhizal data.

Estimating the importance of fixed-effect predictors using likelihood

For likelihood model selection, we fit phylogenetic mixed-effect meta-analysis models with the *rma.mv()* function from version 2.1-0 of the *metafor* package[18] in Microsoft R Open version 3.2.5 in tandem with the Intel® Math Kernel Library. We used the *glmulti()* function from the *glmulti* package[19] version 1.0.7 to automate fitting of all possible models containing different subsets of the candidate fixed-effect predictors. Theory suggests that comparison of information

criteria among models differing in fixed effects is only valid using maximum likelihood (ML) and not restricted maximum likelihood (REML) estimation, but previous studies[20] suggest that REML (and especially the reduced $REML_2$ function[20]) may outperform ML for mixed model selection under a wide range of data structures. So, we conducted model selection using AICc calculated with both ML and $REML_2$, but the primary results presented are those from $REML_2$ model selection; results from ML model selection are also provided for comparison. Results from these model selection analyses were summarized by examining the relative variable importance (RVI) for each fixed-effect predictor, calculated for each predictor as the sum of Akaike weights for models containing that predictor[21]. Predictors with RVI near or below 0.5 were considered to be unimportant in explaining variation in effect size.

Parameter estimates for important fixed effects (all of which were categorical) were calculated as marginal means using the *predict()* function of *metafor* applied to the best models in which those factors occurred. Those best models were also used to estimate the proportion of variance in effect size accounted for by fixed effects, using the marginal R^2 as described by Nakagawa and Schielzeth[22]. The potential for publication bias in our meta-analysis results was assessed by examination of funnel plots of residuals versus their standard errors from the best likelihood models of both the AM-full and EM data sets, using the *funnel()* function of the R package *metafor*.

Bayesian model fitting

To check the sensitivity of results to the model fitting approach, we also used Bayesian approaches to estimate the significance of fixed effects and magnitudes of random effects. We used Markov chain Monte Carlo estimation with the *MCMCglmm()* function of the *MCMCglmm*

package[23] version 2.22.1 in R version 3.2.5. For random effects in these models, we used V (expected (co)variances) = 1 and nu (degree of belief) = 0.002, approximating an inverse gamma prior[24,25]. Bayesian P-values for fixed effects were calculated as the numbers of iterations when one level is greater or less than the other divided by the total number of iterations. These P-values were calculated for fixed effects of each level of categorical variables relative to an intercept representing the estimate across baseline levels of each fixed effect, using models containing all candidate fixed effects. For estimation of random effects, to facilitate direct comparison with results from likelihood model fitting, we used the same fixed effects as in the reduced likelihood models, i.e., those determined to be important using likelihood model selection. For AM-sub and AM-full, this was a model with no fixed effects. For EM, this model contained the fixed effects of Sterilization, N-fertilization, P-fertilization, and Microbial Control. We fit these models as described above and examined posterior mean estimates for the random effects, along with their 95% lower (lower CI) and upper (upper CI) credible intervals.

Alternative imputation methods for missing SD values

To check the robustness of our results to the approach we used for imputation of missing SD values, which were used in calculating variance estimates associated with each effect size estimate, we additionally fit models using two alternative imputation methods. Specifically, we used the *impute_SD* function in the *metagear* package of R to implement its 'Bracken1992'[26] and 'HotDeck_NN'[27] methods; the latter is a multiple imputation method, for which we used M=10, i.e., ten replicate imputed data sets. We applied these imputation methods separately to impute missing SD values in control and mycorrhizal inoculated treatments in both the EM and AM-sub data sets, used those imputed SD values to calculate the estimated variance of each effect size

(log response ratio) estimate using Equation 1 from Hedges et al.[28] and used both REML and Bayesian approaches (as described earlier) to fit meta-analysis models. Both imputation methods, but especially HotDeck_NN, produced imputed SD values with a large range, much larger than in the original data, resulting in some extreme values of estimated variance that were five orders of magnitude larger than the median values of estimated variance. This wide range in variance estimates frequently caused likelihood model fitting to fail. Thus, we removed one observation (out of 2398) from the AM-sub data and three (out of 1001) from the EM data that consistently generated these extreme outliers in estimated variance, and carried out HotDeck_NN imputation on the resulting data. As a result, likelihood model fitting was successful for the EM data, but still typically failed for the arbuscular mycorrhizal data. When likelihood model fitting did not fail, variance component estimates were highly consistent with those from Bayesian model fitting and with our original results, so we only present results from Bayesian (and not likelihood) model fitting. Means and standard errors for variance component estimates from HotDeck_NN imputation were calculated from the 10 replicate estimates according to Rubin's rules[29]. We fit both saturated (with all fixed effects) and reduced (with the same fixed effects as in the best models from the primary analysis) models; although the results were very similar, we present both, to illustrate the minimal effect of fixed-effect structure on variance component estimates, and to facilitate direct comparison with the primary results.

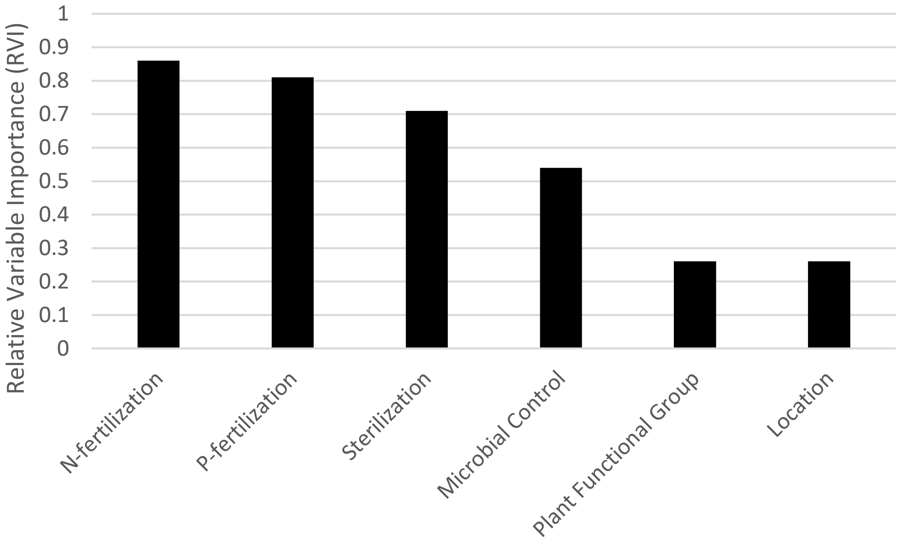

Supplementary Figure 1. Relative variable importance (RVI) of the 6 fixed-effect predictors from maximum likelihood (ML) model selection on meta-analysis models of the ectomycorrhizal (EM) symbiosis data (n=1001 observations)

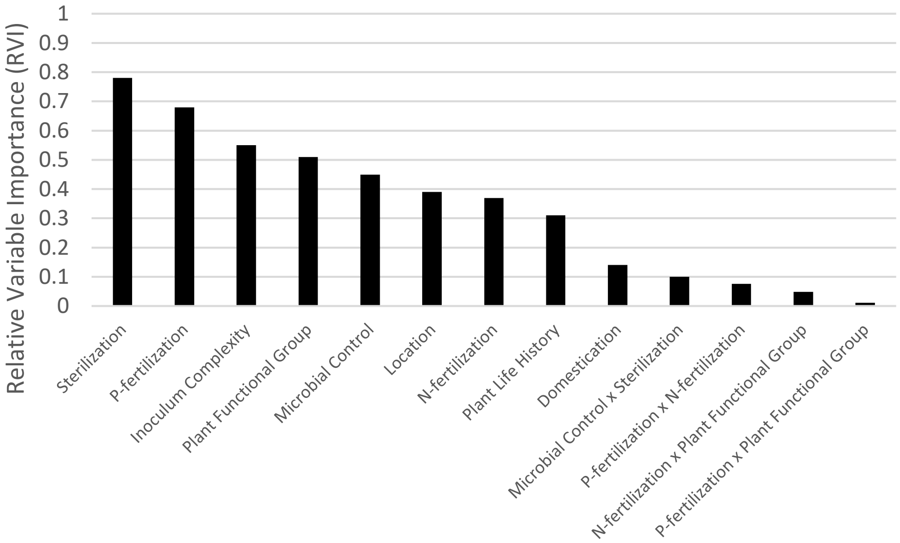

Supplementary Figure 2. Relative variable importance (RVI) of the 13 fixed-effect predictors from maximum likelihood (ML) model selection on meta-analysis models of the AM-full arbuscular mycorrhizal (AM) symbiosis data (n=2984 observations)

Supplementary Table 1. Random-effect variance component estimates from meta-analysis models of arbuscular mycorrhizal (AM) and ectomycorrhizal (EM) symbioses, including results from AM-full data for comparison with AM-sub, and including Bayesian estimates for comparison with likelihood estimates. Likelihood results shown here for AM-sub and EM are identical to those found in Table 1.

Source	Arbuscular Mycorrhiza				Ectomycorrhiza	
	AM-sub data (n=2398)		AM-full data (n=2984)		EM data (n=1001)	
	Likelihood (95% CI*)	Bayesian (95% CI)	Likelihood (95% CI)	Bayesian (95% CI)	Likelihood (95% CI)	Bayesian (95% CI)
Plant Phylogeny	0.009 (0.0-0.15)	0.04 (0.0002-0.14)	**0.10 (0.0 - 0.30), R^2=0.14**	**0.10 (0.0002 - 0.29)**	0.0 (0.0 - 0.07)	0.02 (0.0002 - 0.06)
Plant Species	**0.15 (0.04-0.25), R^2=0.24†**	**0.12 (0.0008-0.21)**	**0.16 (0.02-0.31), R^2=0.22**	**0.16 (0.0006 - 0.28)**	0.0 (0.0 - 0.06)	0.01 (0.0002 - 0.04)
Fungal Phylogeny	0.0 (0.0-0.02)	0.007 (0.0001 - 0.02)	N/A	N/A	0.0 (0.0-0.03)	0.007 (0.0001 - 0.02)
Fungal Genus	0.0 (0.0-0.01)	0.004 (0.0002 - 0.01)	N/A	N/A	0.0 (0.0 - 0.02)	0.005 (0.0001 - 0.02)
Plant Origin	N/A	N/A	N/A	N/A	**0.232 (0.01 - 1.5), R^2=0.18**	**0.30 (0.0002 - 1.0)**
Fungal Origin	N/A	N/A	N/A	N/A	0.0 (0.0 - 0.03)	0.006 (0.0002 - 0.02)
Plant × Fungal Origin	N/A	N/A	N/A	N/A	0.01 (0.0 - 0.05)	0.01 (0.0002 - 0.04)
Plant Phylogeny × Fungal Phylogeny	0.0 (0.0 - 0.06)	0.01 (0.0002 - 0.05)	N/A	N/A	**0.11 (0.01 - 0.16), R^2=0.09**	**0.06 (0.0003 - 0.12)**
Plant Phylogeny × Fungal Genus	**0.06 (0.0-0.09), R^2=0.09**	**0.02 (0.0002 - 0.06)**	N/A	N/A	0.0 (0.0 - 0.05)	0.02 (0.0002 - 0.06)
Plant Species × Fungal Phylogeny	0.0 (0.0-0.05)	0.02 (0.0002 - 0.06)	N/A	N/A	0.0 (0.0 - 0.09)	0.02 (0.0002 - 0.08)
Plant Species × Fungal Genus	0.0001 (0.0-0.06)	0.02 (0.0002 - 0.05)	N/A	N/A	0.0 (0.0 - 0.03)	0.009 (0.0001 - 0.03)
Study ID	**0.10 (0.09-0.11), R^2=0.15**	**0.05 (0.0002-0.10)**	**0.12 (0.11-0.14), R^2=0.17**	**0.03 (0.0003 - 0.11)**	0.04 (0.03-0.05)	0.02 (0.0003 - 0.04)
Control Set	**0.16 (0.14-0.18),**	**0.16 (0.14-0.18)**	**0.14 (0.12-0.17),**	**0.14 (0.12 -**	**0.15 (0.12-0.19),**	**0.15 (0.12 -**

	**R^2=0.24**		**R^2=0.20**	**0.17)**	**R^2=0.12**	**0.19)**
Paper	**0.15 (0.11-0.21), R^2=0.24**	**0.16 (0.11-0.21)**	**0.18 (0.14-0.23), R^2=0.25**	**0.18 (0.14-0.23)**	**0.65 (0.45-0.97), R^2=0.51**	**0.68 (0.44 - 0.96)**

* 95% CI is a profile likelihood confidence interval (likelihood) or credible interval (Bayesian).

† R^2 is a partial conditional R^2, modified from Nakagawa and Schielzeth's[30] conditional R^2 to give proportion of between-studies variance in effect size explained by a particular random effect. Bold print highlights likelihood variance components accounting for more than 5% of between-studies variance in likelihood analysis, for which R^2 is shown.

Supplementary Table 2. Bayesian estimates of **r**andom-effect variance components from meta-analysis models, using two alternative methods for imputing missing SD values before calculating effect size variances (Bracken1992 and HotDeck_NN from the *impute_SD* function of the *metagear* package of R). The same variance components that are in bold in Tables 1 and S1 are bolded here for ease of comparison. Parenthetical uncertainty values are 95% credible intervals for Bracken1992 imputation and posterior standard error for HotDeck_NN imputation.

	AM-sub data (n=2398)				EM data (n=1001)			
	Bracken1992		HotDeck_NN		Bracken1992		HotDeck_NN	
	Saturated model	Reduced model	Saturated model	Reduced model	Saturated model	Reduced model	Saturated model	Reduced model
Plant Phylogeny	0.03 (0.0002-0.11)	0.04 (0.0002-0.13)	0.02 (0.03)	0.03 (0.03)	0.01 (0.0002-0.05)	0.01 (0.0002-0.05)	0.04 (0.04)	0.04 (0.04)
Plant Species	**0.08 (0.0005-0.16)**	**0.11 (0.0004-0.19)**	**0.05 (0.04)**	**0.07 (0.04)**	0.01 (0.0002-0.04)	0.01 (0.0002-0.05)	0.03 (0.03)	0.03 (0.03)
Fungal Phylogeny	0.007 (0.0002-0.02)	0.007 (0.0002-0.02)	0.006 (0.009)	0.006 (0.008)	0.007 (0.0002-0.02)	0.007 (0.0002-0.02)	0.005 (0.005)	0.005 (0.005)
Fungal Genus	0.005 (0.0002-0.01)	0.004 (0.0002-0.01)	0.004 (0.005)	0.004 (0.005)	0.005 (0.0002-0.02)	0.005 (0.0002-0.02)	0.004 (0.004)	0.004 (0.004)
Plant Origin	N/A	N/A	N/A	N/A	**0.33 (0.0002-1.12)**	**0.30 (0.0002-1.10)**	**0.30 (0.42)**	**0.27 (0.39)**
Fungal Origin	N/A	N/A	N/A	N/A	0.007 (0.0001-0.02)	0.007 (0.0001-0.02)	0.005 (0.005)	0.005 (0.005)
Plant × Fungal Origin	N/A	N/A	N/A	N/A	0.01 (0.0002-0.04)	0.01 (0.0002-0.04)	0.009 (0.008)	0.009 (0.008)
Plant Phylogeny × Fungal Phylogeny	0.016 (0.0002-0.05)	0.02 (0.0002-0.06)	0.01 (0.02)	0.01 (0.02)	**0.06 (0.0002-0.12)**	**0.06 (0.0002-0.12)**	**0.01 (0.02)**	**0.01 (0.02)**
Plant	**0.03**	**0.03**	**0.02 (0.01)**	**0.02 (0.02)**	0.02	0.02	0.01 (0.01)	0.01 (0.01)

Phylogeny × Fungal Genus	**(0.0005-0.06)**	**(0.0005-0.06)**			(0.0002-0.06)	(0.0001-0.06)		
Plant Species × Fungal Phylogeny	0.01 (0.0002-0.03)	0.01 (0.0002-0.04)	0.01 (0.02	0.01 (0.02)	0.02 (0.0002-0.07)	0.02 (0.0002-0.07)	0.008 (0.01)	0.009 (0.01)
Plant Species × Fungal Genus	0.02 (0.0003-0.05)	0.02 (0.0003-0.05)	0.02 (0.01)	0.02 (0.01)	0.009 (0.0002-0.03)	0.009 (0.0002-0.04)	0.007 (0.008)	0.007 (0.008)
Study ID	**0.12 (0.04-0.15)**	**0.06 (0.0005-0.13)**	**0.04 (0.03)**	**0.03 (0.03)**	0.03 (0.0005-0.05)	0.02 (0.0005-0.05)	0.02 (0.01)	0.02 (0.01)
Control Set	**0.16 (0.14-0.18)**	**0.16 (0.14-0.19)**	**0.15 (0.01)**	**0.15 (0.01)**	**0.16 (0.12-0.19)**	**0.16 (0.12-0.19)**	**0.13 (0.02)**	**0.13 (0.02)**
Paper	**0.18 (0.12-24)**	**0.16 (0.11-0.21)**	**0.15 (0.03)**	**0.15 (0.02)**	**0.67 (0.42-0.94)**	**0.66 (0.42-0.93)**	**0.05 (0.03)**	**0.05 (0.03)**

Supplementary References

1. Koziol, L. and Bever, J. D. Mycorrhizal response trades off with plant growth rate and increases with plant successional status. *Ecology* **96**, 1768-1774 (2015).
2. Hibbett, D. S. and Matheny, P. B. The relative ages of ectomycorrhizal mushrooms and their plant hosts estimated using Bayesian relaxed molecular clock analyses. *BMC Biology* **7**, 13 (2009).
3. Maherali, H., Oberle, B., Stevens, P. F., Cornwell, W. K., and McGlinn, D. J. Mutualism persistence and abandonment during the evolution of the mycorrhizal symbiosis. *The American Naturalist* **188**, E113-E125 (2016).
4. Tedersoo, L., May, T. W., and Smith, M. E. Ectomycorrhizal lifestyle in fungi: global diversity, distribution, and evolution of phylogenetic lineages. *Mycorrhiza* **20**, 217-263 (2010).
5. Garnica, S., Weiss, M., Walther, G., and Oberwinkler, F. Reconstructing the evolution of agarics from nuclear gene sequences and basidiospore ultrastructure. *Mycological Research* **111**, 1019-1029 (2007).
6. Peintner, U. *et al.* Multiple origins of sequestrate fungi related to *Cortinarius* (Cortinariaceae). *American Journal of Botany* **88**, 2168-2179 (2001).
7. Saar, I., Põldmaa, K., and Kõljalg, U. The phylogeny and taxonomy of genera Cystoderma and Cystodermella (Agaricales) based on nuclear ITS and LSU sequences. *Mycological Progress* **8**, 59-73 (2009).
8. Kohler, A. *et al.* Convergent losses of decay mechanisms and rapid turnover of symbiosis genes in mycorrhizal mutualists. *Nature Genetics* **47**, 410-415 (2015).
9. Matheny, P. B. *et al.* Major clades of Agaricales: a multilocus phylogenetic overview. *Mycologia* **98**, 982-995 (2006).
10. Binder, M. and Hibbett, D. S. Molecular systematics and biological diversification of Boletales. *Mycologia* **98**, 971-981 (2006).
11. Giachini, A. J., Hosaka, K., Nouhra, E., Spatafora, J., and Trappe, J. M. Phylogenetic relationships of the Gomphales based on nuc-25S-rDNA, mit-12S-rDNA, and mit-atp6-DNA combined sequences. *Fungal biology* **114**, 224-234 (2010).
12. Hansen, K. and Pfister, D. H. Systematics of the Pezizomycetes--the operculate discomycetes. *Mycologia* **98**, 1029-1040 (2006).
13. Hosaka, K. *et al.* Molecular phylogenetics of the gomphoid-phalloid fungi with an establishment of the new subclass Phallomycetidae and two new orders. *Mycologia* **98**, 949-959 (2006).
14. Hosaka, K., Castellano, M. A., and Spatafora, J. W. Biogeography of Hysterangiales (Phallomycetidae, Basidiomycota). *Mycological Research* **112**, 448-462 (2008).
15. Methven, A. S., Zelski, S. E., and Miller, A. N. A molecular phylogenetic assessment of the genus Gyromitra in North America. *Mycologia* **105**, 1306-1314 (2013).
16. Nuhn, M. E., Binder, M., Taylor, A. F. S., Halling, R. E., and Hibbett, D. S. Phylogenetic overview of the Boletineae. *Fungal biology* **117**, 479-511 (2013).
17. Wilson, A. W., Binder, M., and Hibbett, D. S. Diversity and evolution of ectomycorrhizal host associations in the Sclerodermatineae (Boletales, Basidiomycota). *New Phytologist* **194**, 1079-1095 (2012).
18. Viechtbauer, W. Conducting meta-analyses in R with the metafor package. *Journal of Statistical Software* **36**, 1-48 (2010).

19. Calgagno, V. glmulti: Model selection and multimodel inference made easy. R package version 1.0.7 (2013).
20. Gurka, M. J. Selecting the Best Linear Mixed Model Under REML. *The American Statistician* **60**, 19-26 (2006).
21. Burnham, K. P. and Anderson, D. R., *Model selection and multimodel inference: A practical information-theoretic approach*. (Springer Science + Business Media, LLC, 2002).
22. Nakagawa, S. and Schielzeth, H. A general and simple method for obtaining R2 from generalized linear mixedâ€• effects models. *Methods in Ecology and Evolution* **4**, 133-142 (2013).
23. Hadfield, J. D. MCMC Methods for Multi-Response Generalized Linear Mixed Models: The MCMCglmm R Package. *Journal of Statistical Software* **33**, 1-22 (2010).
24. Cornwallis, C. K., West, S. A., Davis, K. E., and Griffin, A. S. Promiscuity and the evolutionary transition to complex societies. *Nature* **466**, 969-972 (2010).
25. Horváthová, T., Nakagawa, S., and Uller, T. Strategic female reproductive investment in response to male attractiveness in birds. *Proceedings of the Royal Society B: Biological Sciences* **279**, 163-170 (2011).
26. Bracken, M. B. Statistical methods for analysis of effects of treatment in overviews of randomized trials in *Effective care of the newborn infant* (ed. J. C. Sinclair and M. B. Bracken) 13-20 (Oxford University Press, 1992).
27. Rubin, D. B. and Schenker, N. Multiple imputation in healthcare databases: An overview and some applications. *Statistics in Medicine* **10**, 585-598 (1991).
28. Hedges, L. V., Gurevitch, J., and Curtis, P. S. The meta-analysis of response ratios in experimental ecology. *Ecology* **80**, 1150-1156 (1999).
29. Lajeunesse, M. J. Recovering missing or partial data from studies: a survey of conversions and imputations for meta-analysis in *Handbook of meta-analysis in ecology and evolution* (ed. J. Koricheva, J. Gurevitch, and K. Mengersen) 195−206 (Princeton University Press, 2013).
30. Nakagawa, S. and Schielzeth, H. A general and simple method for obtaining R2 from generalized linear mixed-effects models. *Methods in Ecology and Evolution* **4**, 133-142 (2013).
